# Investigation of Microstructure and Mechanical Properties for Ti-6Al-4V Alloy Parts Produced Using Non-Spherical Precursor Powder by Laser Powder Bed Fusion

**DOI:** 10.3390/ma14113028

**Published:** 2021-06-02

**Authors:** Jaime Varela, Edel Arrieta, Muktesh Paliwal, Mike Marucci, Jose H. Sandoval, Jose A. Gonzalez, Brandon McWilliams, Lawrence E. Murr, Ryan B. Wicker, Francisco Medina

**Affiliations:** 1 Department of Mechanical Engineering, The University of Texas at El Paso, El Paso, TX 79938, USA; jvarela11@miners.utep.edu (J.V.); egarrieta@utep.edu (E.A.); rwicker@utep.edu (R.B.W.); 2 W.M. Keck Center for 3D Innovation, University of Texas at El Paso, El Paso, TX 79968, USA; lemurr@utep.edu; 3 Kymera International—Reading Alloys, Robesonia, PA 19551, USA; muktesh.paliwal@kymerainternational.com (M.P.); mike.marucci@kymerainternational.com (M.M.); 4 Lockheed Martin Missiles and Fire Control, Dallas, TX 75051, USA; jose.h.sandoval@lmco.com (J.H.S.); jose.a1.gonzalez@lmco.com (J.A.G.); 5 CCDC Army Research Laboratory, Aberdeen Proving Ground, MD 21005, USA; brandon.a.mcwilliams.civ@mail.mil; 6 Department of Metallurgical, Materials and Biomedical Engineering, The University of Texas at El Paso, El Paso, TX 79968, USA

**Keywords:** non-spherical, hydride-dehydride (HDH) Ti-6Al-4V powder, laser powder bed fusion, post-process heat treatment, microstructure, mechanical properties

## Abstract

An unmodified, non-spherical, hydride-dehydride (HDH) Ti-6Al-4V powder having a substantial economic advantage over spherical, atomized Ti-6Al-4V alloy powder was used to fabricate a range of test components and aerospace-related products utilizing laser beam powder-bed fusion processing. The as-built products, utilizing optimized processing parameters, had a Rockwell-C scale (HRC) hardness of 44.6. Following heat treatments which included annealing at 704 °C, HIP at ~926 °C (average), and HIP + anneal, the HRC hardnesses were observed to be 43.9, 40.7, and 40.4, respectively. The corresponding tensile yield stress, UTS, and elongation for these heat treatments averaged 1.19 GPa, 1.22 GPa, 8.7%; 1.03 GPa, 1.08 GPa, 16.7%; 1.04 GPa, 1.09 GPa, 16.1%, respectively. The HIP yield strength and elongation of 1.03 GPa and 16.7% are comparable to the best commercial, wrought Ti-6Al-4V products. The corresponding HIP component microstructures consisted of elongated small grains (~125 microns diameter) containing fine, alpha/beta lamellae.

## 1. Introduction

Additive manufacturing/3D printing has become a key enabling manufacturing process which has been characterized as underpinning the so-called Fourth Industrial Revolution. Powder-bed fusion processes such as selective laser melting (SLM) and electron beam melting (EBM) in particular provide a wide range of cost savings, high precision and speed of production for complex product shapes applied to aerospace automotive, biomedical and related applications, including maintenance, repair and sustainment. Qualification and certification of optimized parts, especially aerospace and aircraft components are also important issues [1,2,3,4,5,6].

Due to its low density, high mechanical strength, excellent corrosion resistance, and related properties, Ti-6Al-4V alloy has become one of the most widely used titanium alloys for additive manufacturing of a wide range of components [7,8], especially for the laser-based or laser beam powder-bed fusion (LBPBF) process. Since the laser-based (SLM) process utilizes an inert gas atmosphere (argon or nitrogen), this shield gas flow reduces oxidation of the alloy powder and the melted layers. However, the morphology, size and size distribution of the precursor powder are also important factors because they affect powder flowability, laser beam energy absorption, and conductivity of the powder bed which change as the bed consolidates and melts. In addition, the laser process parameters have a controlling effect on layer building as well as the microstructure and properties of the as-built product. These generally include laser beam energy and energy density which is related to the absorbed energy in the powder layer, the scan speed, and the beam size or scan spacing [8,9].

It is apparent, as noted above, that the initial powder bed particle packing or packing density has an effect on the laser beam energy absorption and melt efficiency of the layers, and this will in fact change as the powder bed layer melts. While spherical powders having a wider distribution of particle sizes can optimize bed packing and densification by more effectively filling void spaces with smaller particles requisite flowability, these powders generally have a high cost since their production involves gas or plasma atomization [9]. In contrast, Ti-6Al-4V powders having non-spherical shapes are easily produced by forming stable, brittle hydrides that can be crushed, milled, and screened to produce fine powders which are dehydrided to form non-spherical alloy powder. This hydride-dehydride (HDH) process is a long-established process for Ti and Ti alloy powder production [10,11], and represents a significant economic advantage over spherical, atomized powder production [12,13,14].

The challenge for powder bed fusion fabrication utilizing non-spherical precursor powder is the achievement of requisite flowability, packing, and melt efficiency in order to achieve optimized part production characterized by short production times to achieve requisite mechanical properties and associated microstructures. Jaber et al. [12] have recently demonstrated that for L-PBF of a hybrid-50% spherical and 50% non-spherical (HDH)-Ti-6Al-4V powder, the flowability was the crucial parameter governing the residual tensile properties of fabricated components. Hou et al. [15] also recently modified HDH Ti-6Al-4V powder by ball milling and were able to produce 99% dense products for this modified powder using laser beam powder-bed fusion processing. Narra et al. [16] have also compared melt pool porosity for electron beam powder-bed fusion processing of spherical and non-spherical Ti-6Al-4V alloy powders. Microstructures observed for HDH Ti-6Al-4V alloy builds were observed to be similar to those for parts fabricated using spherical, atomized powders.

In this investigation, laser beam powder-bed fusion process parameters were systematically varied in order to find optimized conditions to fabricate an assortment of complex Ti-6Al-4V alloy products and test components; utilizing 100% non-spherical HDH Ti-6Al-4V alloy powder. While the as-fabricated components were near full density, conventional post-process annealing relieved process-induced internal stress, while HIP reduced remaining porosity and produced components with microstructures and mechanical properties compatible with those characteristic of Ti-6Al-4V alloy products fabricated using spherical, atomized precursor powder and heat treated. In fact, as-built and post process HIP components fabricated from non-spherical, HDH Ti-6Al-4V alloy powder using laser beam powder-bed fusion exhibited tensile properties as good as the best, commercial Ti-6Al-4V wrought products.

## 2. Materials and Methods

The recent work of Jaber et al. [12] as noted above compared laser beam powder-bed fusion components of Ti-6Al-4V utilizing 100% commercial, spherical powder and a 1-to-1 mixture of spherical and non-spherical (HDH) Ti-6Al-4V alloy powder. The powder mixture exhibited a 30% reduction in flowability while the corresponding, as-built components exhibited porosity and large, unmelted fusion zones ~100 microns in size. The tensile strength and elongation for the mixed powder components were observed to be ~17% and 31% lower, respectively, than for the 100% spherical powder-fabricated components. It is notable that the build process parameters were constant at 125 W beam energy and 10^3^ mm/s laser beam scan speed for a layer thickness of ~20 μm.

### 2.1. Powder and Feedstock

**Figure 1:**
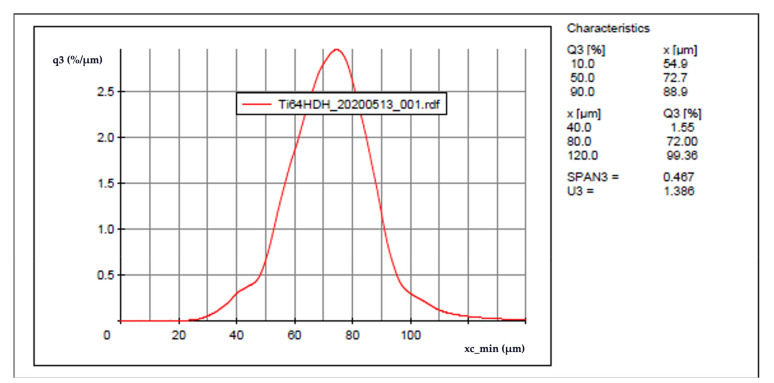
Particle size distribution.

**Figure 2:**
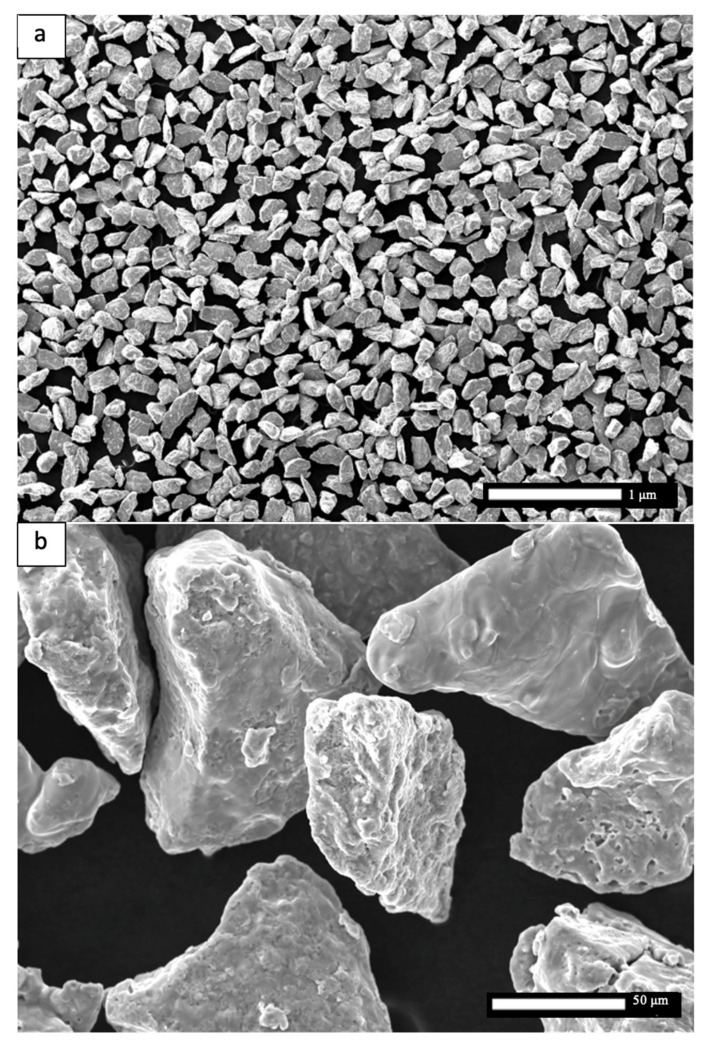
Ti64 HDH powder SEM images. (**a**) Low magnification. (**b**) High magnification.

In the present work, the laser powder-bed fusion process parameters were systematically varied in fabricating simple test blocks or cubes to achieve a wide range of laser beam scan speeds and volumetric energy densities in non-spherical, HDH Ti-6Al-4V alloy powder layers ~30 microns thick. Optimum process parameters were as selected on the basis of minimum, as-built test cube porosities and corresponding maximum densities. Additionally, post-process heat treatments were also utilized to create essentially full density, low porosity products. Unlike the work of Hou et al. [15] utilizing modified (ball-milled) HDH powder Ti-6Al-4V powder in electron beam powder-bed fusion processing, the current study utilized as produced Ti-6Al-4V (Ti64) HDH powder as shown in the SEM images in Figure 1 and provided by Reading Alloys-Kymera International (Raleigh, NC, USA). Particle size and morphology analysis was performed through the Retsch Camsizer X2 (Haan, Germany) Dynamic Image Analysis system. Analysis revealed a particle size distribution of D10: 54.9 μm, D50: 72.7 μm, and D90: 88.9 μm as shown in Figure 2. The scanning electron microscope (SEM) images of the powder shown in Figure 1 were taken with a JEOL JSM-IT500 SEM (Tokyo, Japan).

### 2.2. Process Parameters

Ti-6Al-4V HDH samples were fabricated on a preheated bedplate at 175 °C. A design of experiments was performed to develop the optimal printing parameters for the HDH powder. Fourteen 15 mm × 15 mm × 15 mm test cubes were printed with various parameter changes. A layer thickness of 30 μm and a stripe width of 10 mm were kept constant. Scan speed, laser power, hatch distance all changed to yield different volumetric energy densities as shown below in Table 1.

Cube one yielded the least noticeable porosity from optical micrographs as well as the highest density from pycnometry. These were the chosen printing parameters moving forward.

### 2.3. Laser Powder Bed Fusion System, Setup, and Fabrication

**Figure 3:**
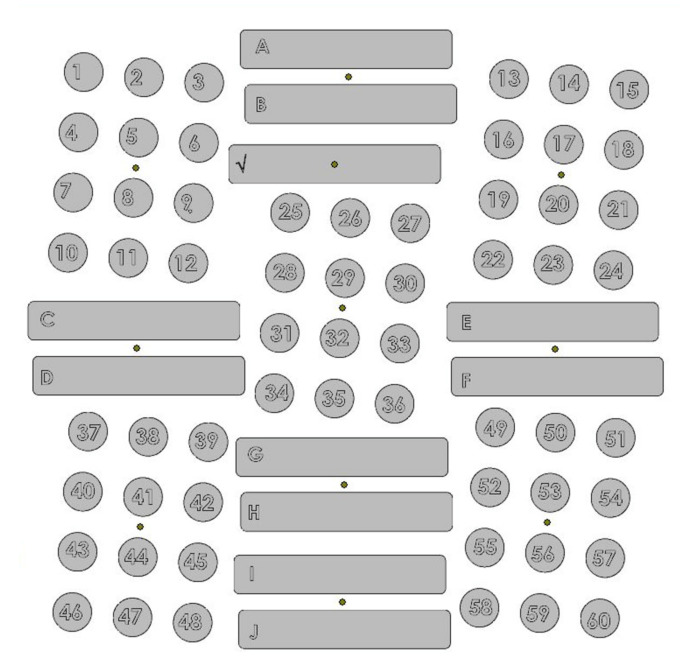
Build plate print layout.

The fabrication of the Ti64 HDH pieces was completed with an EOS M290 (Krailling, Germany). The M290 is an industrial production LPBF system equipped with 400 W Ytterbium fiber laser and a 250 mm × 250 mm × 325 mm build volume. Sixty Ø 14 mm × 80 mm vertical cylinders and eleven 77 mm × 14 mm × 60 mm bars (A–J in Figure 3) were fabricated as shown in the layout in Figure 3. Of the sixty successfully printed cylinders eleven were allocated for the annealing heat treatment described in Section 2.4, six for HIP, and eleven for the combination of HIP and annealing.

**Figure 4:**
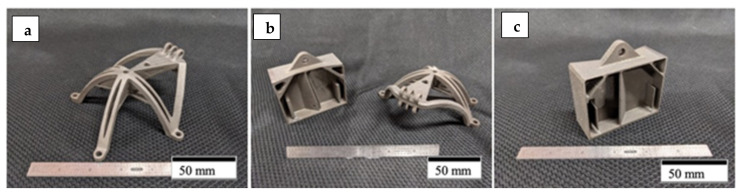
Annealed parts successfully printed from Ti64 HDH. (**a**) Topologically optimized bracket. (**b**) Side to side printed geometries. (**c**) Electronics box enclosure.

**Figure 5:**
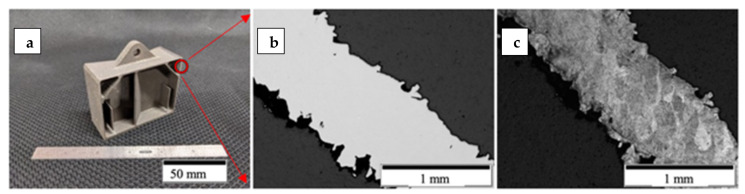
Sectioned printed part. (**a**) Sectioned electronic box enclosure at the red circle. (**b**) Polished micrograph. (**c**) Etched micrograph.

In addition to the various test geometries fabricated as shown in Figure 3, a series of complex, aerospace components were fabricated from the non-spherical HDH Ti64 powder utilizing the optimized process parameters described above. These are illustrated in the examples shown in Figure 4 and Figure 5. Figure 5b,c depicts the internal porosity and microstructure representations, respectively, of the electronics box enclosure section at the indicated red circle (Figure 5a).

The parts fabricated were an electronics box enclosure and a topologically optimized bracket. The bracket and electronics box enclosure were designed by incorporating elements from a Design for Additive Manufacturing (DfAM) perspective and took into account L-PBF process capabilities. Both parts are representative of use cases for Department of Defense and Aerospace industry applications. The parts produced under this program using Ti64 HDH metal powder feedstock was selected as a case study and allow for a one-to-one comparison against the same geometries that were built using Gas Atomized or Plasma Atomized metal powders in L-PBF.

### 2.4. Heat Treatment Parameters

All tensile samples were heat treated prior to machining. The annealing was performed in accordance with the SAE Aerospace AMS 2801B parameters. HIP parameters followed the ASTM F2924-14 Standard. Detailed heat treatment parameters for three variants are shown in Table 2 below.

### 2.5. Tensile Testing

Following the heat treatment process all parts were machined in accordance with ASTM E8 standard. An MTS Landmark (Eden Prairie, MN, USA) servo-hydraulic tensile test system was utilized for monotonic uniaxial tensile strength tests. The testing was performed with threaded grips and an MTS 30 mm axial clip extensometer for axial strain measurements. Samples were strained at a rate of 0.47625 mm/min. Results were averaged from 11 specimens in the Anneal (Variant 1) and the HIP + Anneal (Variant 3); and averaged from six specimens in the HIP variant.

### 2.6. Microstructure Characterization

**Figure 6:**
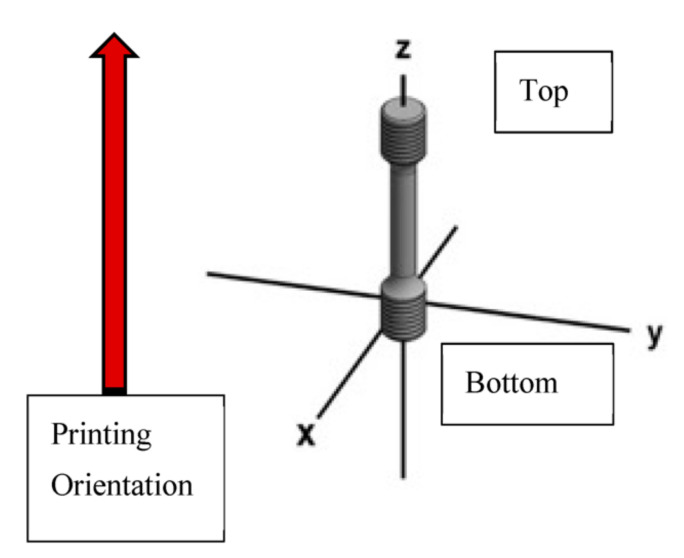
Machined tensile specimen sectioned at the top and bottom threaded sections for metallography.

Each tensile specimen had two threaded sections of an approximate of 12 mm in diameter and 15 mm of length. These unstrained threaded sections were sectioned both at the top and the bottom for metallographic analysis as shown in Figure 6. This method was performed in order to study the sections of the tested samples in a location that was not affected by the tensile test therefore not disrupting the microstructure representation. Each sample was sectioned as to reveal the X, Y, and Z planes with the X and Y planes being in accordance with the printing orientation.

All metallographic samples were created with the ATM OPAL 460 (Haan, Germany) hot mounting press and black epoxy. Metallographic samples were then ground and polished using the ATM SAPHIR 530 (Haan, Germany) semiautomatic system. The grinding process began with Silicon Carbide abrasive paper with 320 grit at 300 rpm with a force of 35 N until plane. Samples were subsequently more finely grinded with a 9 µm diamond suspension on a fine grinding disc at 150 rpms with a force of 25 N for 5 min. The final polishing step was performed on a neoprene polishing pad with a 0.2 µm fumed silica suspension at 150 rpm with a force of 25 N for 5 min.

Microstructure was revealed using Kroll’s Reagent consisting of 92 mL of distilled water, 6 mL of nitric acid and 2 mL of hydrofluoric acid. Samples were submerged in the reagent for 10–15 s depending on the variant. Optical microscopy was performed in an Olympus™ GX53 (Olympus Inc., Tokyo, Japan).

### 2.7. Density Measurements

**Figure 7:**
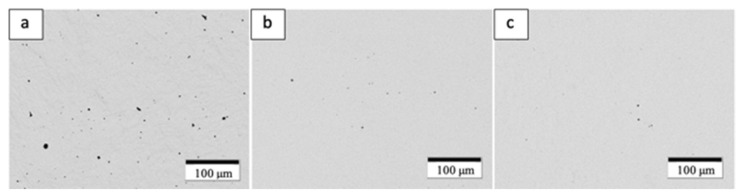
Polished optical micrographs demonstrating porosity across variants. (**a**) Annealed, (**b**) HIP, (**c**) HIP + Annealed.

Gas displacement pycnometry was performed on an AccuPyc II 1340 (Norcross, GA, USA) to obtain volume measurement. The AccuPyc performed five measurements of every sample giving an average. Mass measurements were obtained from a Sartorius CP124S weight balance (Sartorius AG, Göttingen, Germany). Density was then calculated from the resulting volume and mass measurements. In addition, each variant was sectioned and polished to obtain visual representation of internal porosity as demonstrated in Figure 7.

### 2.8. Hardness Testing

Hardness measurements were performed on the Wilson^®^ Rockwell^®^ 2000 (Canton, MA, USA) hardness tester. Samples were indented with a Brale indenter on the Rockwell C scale (HRC). This was done with a pre-load of 10 Kgf and a main-load of 150 Kgf. Measurements were performed on two samples from each variant at the top and bottom portions and the X, Y, and Z surfaces. Three indentations were made on every surface separated by at least one millimeter. An average from all these measurements were then reported.

### 2.9. Fracture Surface Analysis

Following the tensile tests, the fractures of each variant were analyzed using the JEOL JSM-IT500 SEM (JEOL, Tokyo, Japan) scanning electron microscope. One end of the fracture surface from each variant was mounted for observation and comparison.

### 2.10. Chemical Analysis

Chemical analysis was performed by Reading Alloys-Kymera International. O, N, H analysis was performed on an 836 Series Elemental Analyzer from the LECO corporation (St. Joseph, MI, USA). C and S measurements were done on an CS744 Series Carbon/Sulfur Analyzer from LECO (St. Joseph, MI, USA). Al, V, Fe, Si, and Ca results were obtained from the Ciros Vision Inductively Coupled Plasma Spectrometer from SPECTRO Analytical Instruments (Kleve, Germany). Results met grade 5 chemistry for Ti64 as shown in Table 3.

## 3. Results and Discussion

### 3.1. Microstructure Analysis and Discussion

**Figure 8:**
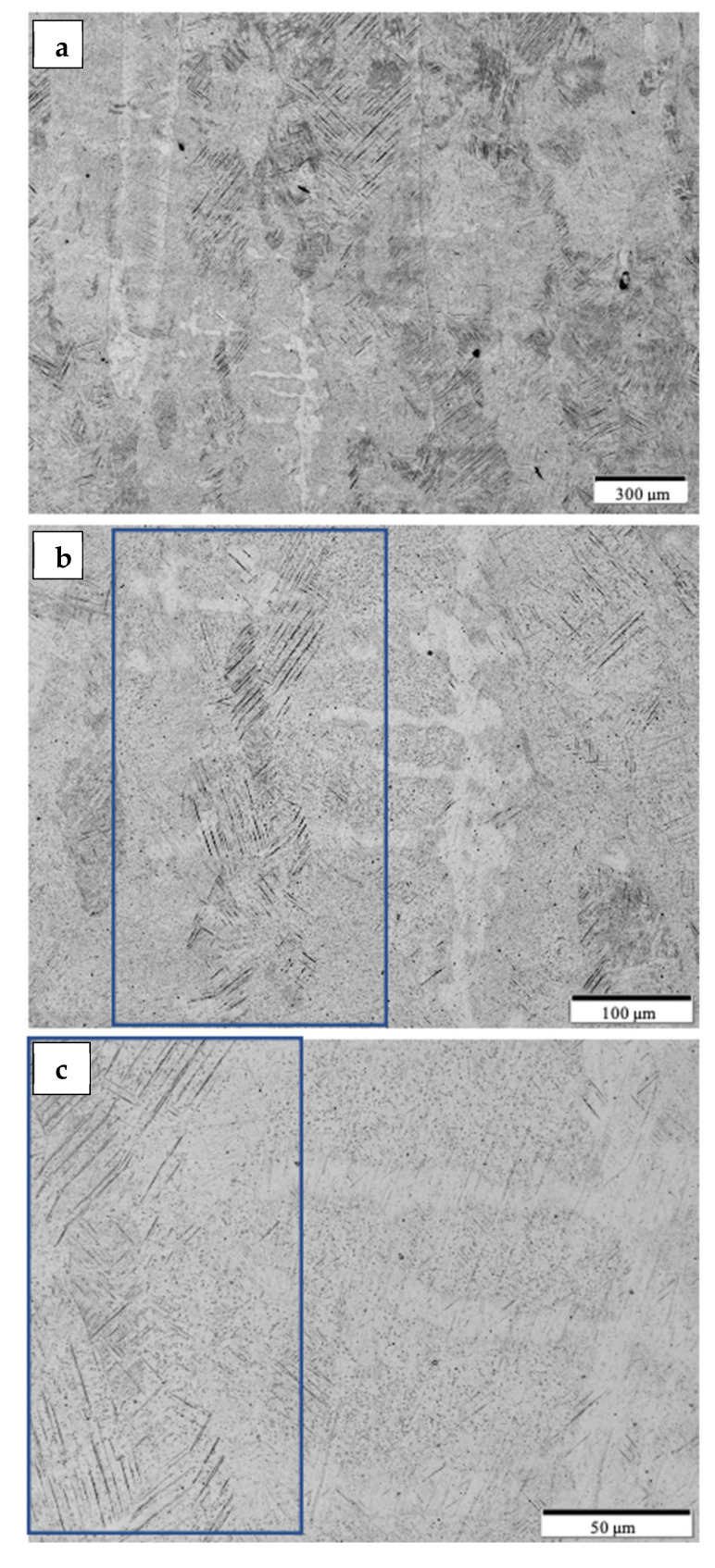
Optical micrographs of as-built DOE cubes from the build middle. (**a**) Low magnification. (**b**) Medium magnification. Alpha-prime enclosed in the blue rectangle. (**c**) High magnification. Alpha-prime enclosed in the blue rectangle.

Figure 8 shows the typical microstructure for the optimized, as-built Ti-6Al-4V alloy product utilizing the non-spherical, HDH powder in Figure 1. The low magnification image in Figure 8a shows varying sizes of grains elongated in the build direction along with layer-related melt bands. Build direction for all micrograph images is oriented from bottom to top. The higher magnification views in Figure 8b,c show a preponderance of alpha-prime martensite represented by the black lamellae which are variously etched in optimally oriented grains. These martensite lamellae, having widths of ~2 microns, result from the rapid cooling associated with the laser beam processing. The corresponding HRC hardness average characteristic of the test components represented in Figure 8 was 44.6, which along with the martensitic microstructure in Figure 8b,c is typical for LPBF as-built Ti-6Al-4V alloy components utilizing spherical, atomized precursor powder [2,7,8,9,17]. The alpha-prime (martensite) microstructure shown in Figure 8b,c results from the diffusionless, composition invariant beta → alpha-prime martensite transformation, and was also observed by Jaber et al. [12] using an HDH-non-spherical/spherical Ti-6Al-4V powder mixture for laser beam powder-bed fusion processing, as well as the more recent work of Narra et al. [16] using a modified, non-spherical HDH Ti-6Al-4V alloy precursor powder. 

**Figure 9:**
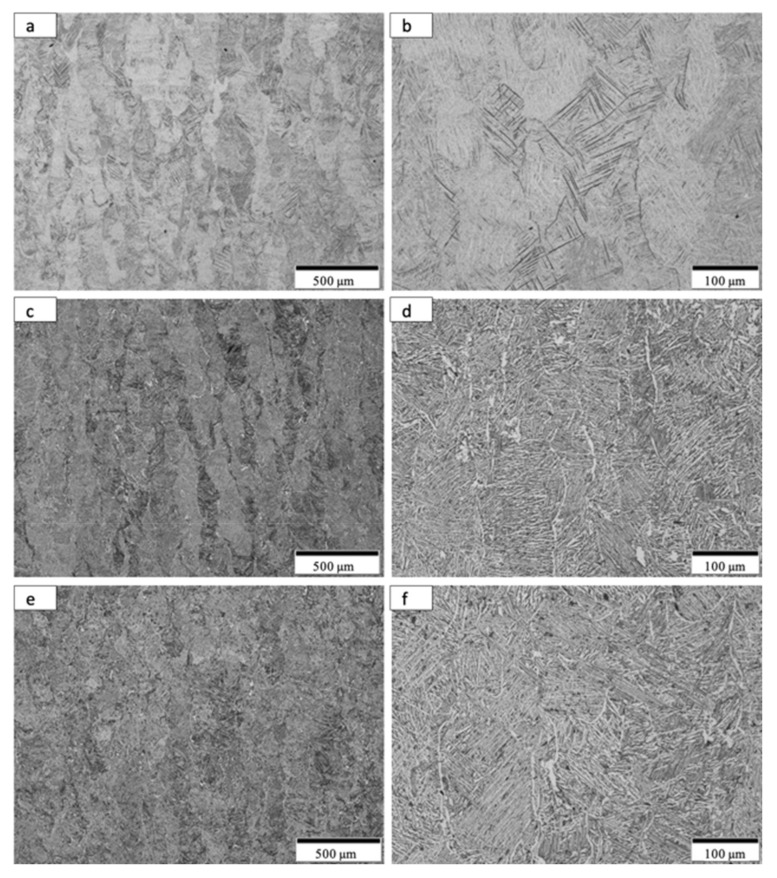
Optical micrographs for Anneal, HIP and HIP + Anneal samples from the build bottom. (**a**,**b**) show low and high magnification images for anneal samples. (**c**,**d**) show low and high magnification images for HIP samples. (**e**,**f**) show low and high magnification image for HIP + Anneal samples.

**Figure 10:**
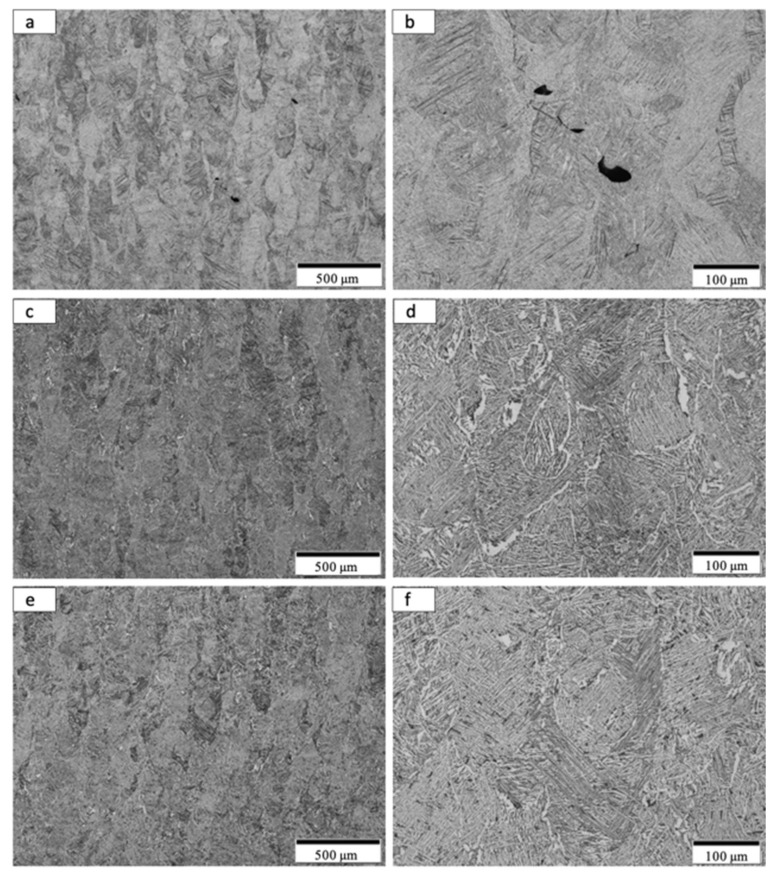
Optical micrographs for Anneal, HIP and HIP + Anneal samples from the build top. (**a**,**b**) show low and high magnification images for anneal samples. (**c**,**d**) show low and high magnification images for HIP samples. (**e**,**f**) show low and high magnification image for HIP + Anneal samples.

By comparison with the as-built microstructure represented typically in Figure 8, the non-spherical, HDH Ti64 as-built and annealed Ti-6Al-4V product microstructure typical for the complex component fabrications shown in Figure 4 and Figure 5 is illustrated in Figure 9 and Figure 10a,b at the bottom and top for test rods (Figure 6), respectively. There is little difference for both the low and high magnification images in Figure 9 and Figure 10a,b in contrast to the corresponding as-built microstructures shown in Figure 8a,c. It is also noted in Figure 10b that there is some residual porosity at the top of the fabricated component where the temperature is highest. The optical micrographs in Figure 9b and Figure 10b show the black, lamellar contrast characteristic of the alpha-prime martensite shown in Figure 8c. The Rockwell C-scale hardness corresponding to Figure 9b and Figure 10b averaged HRC 43.9; this reduction from the as-built component hardness of HRC 44.6 noted above attests to the stress-relief provided by the anneal, which ideally involves the annihilation of process-induced dislocations.

Following HIP of the LPBF as-built Ti-6Al-4V components using the non-spherical HDH precursor powder (Figure 1), the variously elongated grain structure, with average grain sizes of ~125 microns, was essentially unchanged (Figure 9c and Figure 10c) while the black, lamellar (and acicular) alpha-prime martensite shown in Figure 9b and Figure 10b was replaced by varying sizes and distributions of lamellar alpha (alpha/beta), along with non-lamellar and globular alpha which is rendered white in the etching to produce the optical micrographs shown in Figure 9d and Figure 10d. Some very small residual alpha-prime is also observed as tiny black dots. It is also notable that the widths of the lamellar alpha segments are ~2 microns. The corresponding hardness characteristic of the HIP microstructures shown in Figure 9 and Figure 10c,d averaged HRC 40.7; representing a reduction of ~7% from the annealed components noted above. These results are similar to components fabricated from commercial, spherical Ti64 powder by LPBF where the HIPed (at 920 °C) microhardness dropped by ~13% in contrast to as-built and stress-relief annealed (at 704 °C) components [18].

As shown in Figure 9e,f and Figure 10e,f HIPed (at ~926 °C) and annealed (at 704 °C) components exhibited little change in the residual microstructure from the HIPed components (Figure 9c,d and Figure 10c,d). This feature was also attested to by the characteristic, average hardness of HRC 40.4; essentially unchanged from HRC 40.7 for HIPed components.

It can be observed on comparing Figure 9 and Figure 10b,d,f that the lamellar martensite in Figure 9b and Figure 10b, having a thickness of ~2 microns, is essentially unchanged for the transformed, lamellar alpha (alpha/beta) microstructure in Figure 9 and Figure 10d,f. There is also some non-lamellar alpha and globular alpha. This microstructure variation as a consequence of HIP treatment accounts for the hardness variation.

### 3.2. Tensile Testing and Mechanical Property Comparisons and Discussion

**Figure 11:**
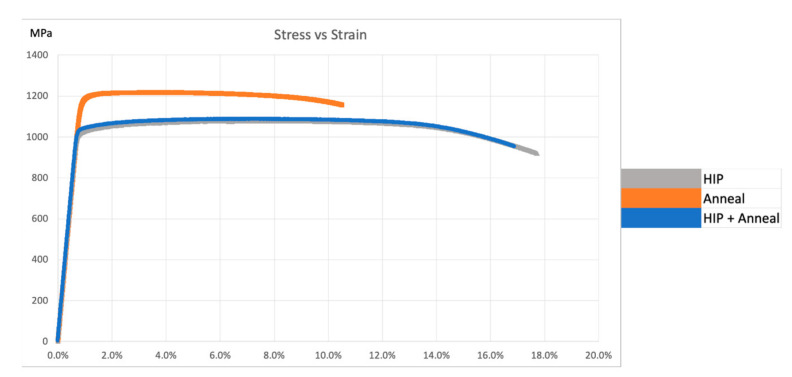
Stress vs. strain curves of individual samples closest to the average.

**Figure 12:**
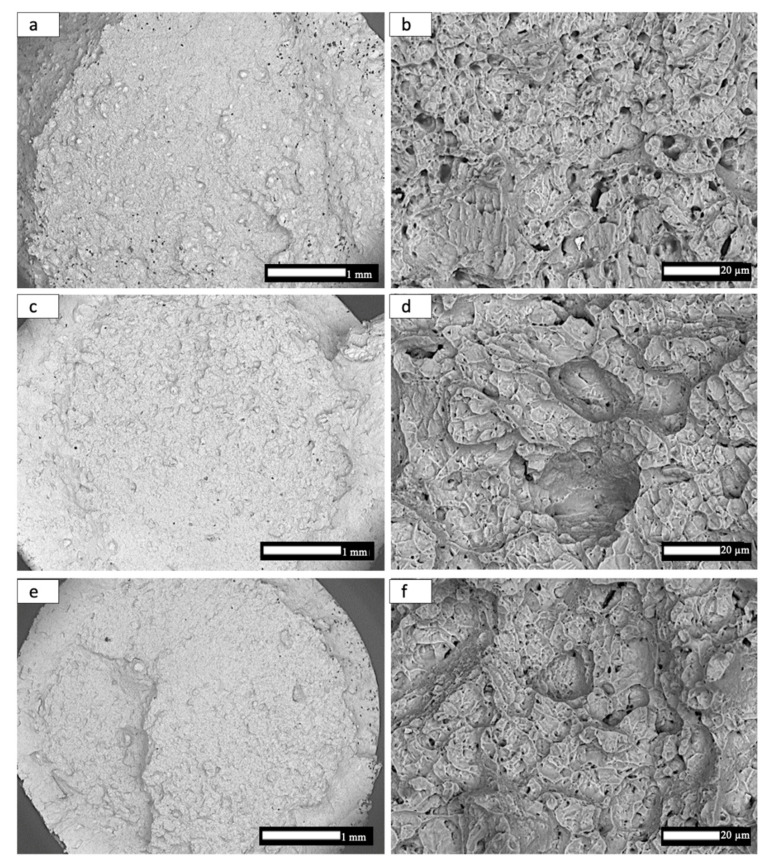
SEM images of fracture surfaces. (**a**,**b**) show low and high magnification of the Anneal fracture. (**c**,**d**) show low and high magnification of the HIP fracture. (**e**,**f**) show low and high magnification of the HIP + Anneal fracture.

The results of tensile tests for the heat-treated, non-spherical HDH Ti-6Al-4V powder-fabricated components, and corresponding to microstructures presented in Figure 9 and Figure 10, are summarized in Table 4. The average yield stress and UTS of ~1.2 GPa and elongation (Ɛmax) of ~8.7% for the annealed components is characteristic of mill-annealed and solution-treated and aged bars and billets of Ti-6Al-4V; as well as laser beam powder-bed fusion fabricated Ti-6Al-4V components using spherical, atomized precursor powder [7,8,9,15,19,20,21,22]. Figure 11 compares tensile stress–strain diagrams for test specimens exhibiting properties closest to the averages shown in Table 4. Table 4 also compares the average component densities and the Rockwell C-scale (HRC) hardnesses. Figure 12 compares the fracture surface features corresponding to the stress–strain diagrams for heat-treated specimens shown in Figure 11. Ductile dimples 1–2 microns in diameter dominate the annealed specimens shown in Figure 12b, while somewhat larger dimple sizes averaging 2–3 microns characterize the HIP and HIP + anneal specimens shown in Figure 12d,f, respectively. These variations in ductile dimple sizes roughly corroborate the ductility values shown in Table 4 and Figure 11. There is also notable porosity associated with the annealed component shown in the image in Figure 10b, and this may contribute to the reduced ductility in contrast to the HIP and HIP + anneal components.

The yield stress, UTS, and elongation associated with the HIPed components (Variant 2 in Table 4) are exceptional and exceed laser powder bed fusion Ti64 components fabricated from traditional, spherical, atomized alloy powders [21,22]. This is due in part to the closing of small pores as shown in Figure 7a,b, and increased density implicit in Table 4. The fine, lamellar alpha/beta microstructure shown in Figure 9d and Figure 10d also facilitates the resulting high yield stress and ductility; which are equivalent to the best commercial, wrought Ti-6Al-4V products.

## 4. Conclusions

Heat treatment of Ti-6Al-4V components, especially HIP of as-built components fabricated from 100% non-spherical, HDH precursor powder by laser beam powder-bed fusion, has produced nearly fully dense, high strength (yield stress > 1 GPa) and high ductility (~17% elongation) products. The resulting mechanical properties rival the best commercial, wrought Ti-6Al-4V products, and exceed Ti-6Al-4V products fabricated by laser powder bed fusion utilizing more conventional, spherical, atomized precursor powders following heat treatment, including HIP. It is especially notable that superior Ti-6Al-4V alloy products have been fabricated using 100% non-spherical, HDH which represents a considerable economic advantage over conventional spherical, atomized precursor powder. This represents a milestone development in Ti-6Al-4V additive manufacturing.

## Figures and Tables

**Table 1:** Process parameters design of experiments.

Cube -	Scan Speed (mm/s)	Laser Power (W)	Hatch (mm)	Layer Thickness (μm)	Stripe Width (mm)	e (J/mm^3^)
1	1200	280	0.14	30	5	55.6
2	1200	310	0.14	30	5	61.5
3	1200	340	0.14	30	5	67.5
4	1100	280	0.14	30	5	60.6
5	1000	280	0.14	30	5	66.7
6	1000	280	0.12	30	5	77.8
7	1000	280	0.1	30	5	93.3
8	400	110	0.12	30	5	76.4
9	400	120	0.12	30	5	83.3
10	400	130	0.12	30	5	90.3
11	360	110	0.12	30	5	84.9
12	320	110	0.12	30	5	95.5
13	320	110	0.1	30	5	114.6
14	320	110	0.08	30	5	143.2

**Table 2:** Hot isostatic pressing and annealing parameters.

Variant	Process	Pressure (MPa)	Temperature (°C)	Hold Time (min)	Cooling
1	Anneal	None	704 ± 14	120 ± 15	Air or furnace cooled
2	HIP	100	896–955 (±15 of selected temp)	180 ± 60	Under inert atmosphere below 425 °C
3	HIP	100	896–955 (±15 of selected temp)	180 ± 60	Under inert atmosphere below 425 °C
	Anneal	None	704 ± 14	120 ± 15	Air or furnace cooled

**Table 3:** Chemical composition of as-built parts.

Ti64 HDH	**Al**	**V**	**Fe**	**Si**	**Ca**	**O**	**N**	**C**	**S**	**H**
	5.92	4.06	0.20	0.014	0.006	0.17	0.042	0.033	0.0009	0.009

**Table 4:** Mechanical properties of tensile samples using HDH Ti-6Al-4V.

Variant	Yield Stress (MPa)		UTS (MPa)		Ɛmax (%)		Density (g/cm^3^)		Relative Density *	Hardness (HRC)	
	Mean	SD **	Mean	SD **	Mean	SD **	Mean	SD **	Mean	Mean	SD **
Anneal	1185	15	1217	13	8.7	1.3	4.39	0.004	99.2%	43.9	1.36
HIP	1025	17	1083	13	16.7	0.8	4.41	0.006	99.5%	40.7	0.93
HIP + Anneal	1039	9	1089	9	16.1	1.1	4.40	0.005	99.4%	40.4	0.97
As-built	--	--	--	--	--	--	4.38	0.002	98.9%	44.6	0.71

* 4.43 g/cm^3^ as full density. ** Standard deviation.

## Data Availability

Not applicable.
